# Hsp110 Chaperones Regulate Prion Formation and Propagation in *S. cerevisiae* by Two Discrete Activities

**DOI:** 10.1371/journal.pone.0001763

**Published:** 2008-03-12

**Authors:** Heather Sadlish, Heike Rampelt, James Shorter, Renee D. Wegrzyn, Claes Andréasson, Susan Lindquist, Bernd Bukau

**Affiliations:** 1 Zentrum für Molekulare Biologie der Universität Heidelberg (ZMBH), DKFZ-ZMBH Alliance, Universität Heidelberg, Heidelberg, Germany; 2 Department of Biochemistry and Biophysics, University of Pennsylvania School of Medicine, Philadelphia, Pennsylvania, United States of America; 3 Howard Hughes Medical Institute, Whitehead Institute for Biomedical Research, Cambridge, Massachusetts, United States of America; Massachusetts Institute of Technology, United States of America

## Abstract

The cytosolic chaperone network of *Saccharomyces cerevisiae* is intimately associated with the emergence and maintenance of prion traits. Recently, the Hsp110 protein, Sse1, has been identified as a nucleotide exchange factor (NEF) for both cytosolic Hsp70 chaperone family members, Ssa1 and Ssb1. We have investigated the role of Sse1 in the *de novo* formation and propagation of [*PSI* ^+^], the prion form of the translation termination factor, Sup35. As observed by others, we find that Sse1 is essential for efficient prion propagation. Our results suggest that the NEF activity is required for maintaining sufficient levels of substrate-free Ssa1. However, Sse1 exhibits an additional NEF-independent activity; it stimulates *in vitro* nucleation of Sup35NM, the prion domain of Sup35. We also observe that high levels of Sse1, but not of an unrelated NEF, very potently inhibit Hsp104-mediated curing of [*PSI* ^+^]. Taken together, these results suggest a chaperone-like activity of Sse1 that assists in stabilization of early folding intermediates of the Sup35 prion conformation. This activity is not essential for prion formation under conditions of Sup35 overproduction, however, it may be relevant for spontaneous [*PSI* ^+^] formation as well as for protection of the prion trait upon physiological Hsp104 induction.

## Introduction

Prions are infectious conformers of proteins that can convert the natively folded species to the prion form. The yeast *Saccharomyces cerevisiae* carries epigenetic determinants that behave as prions by adopting a conformation capable of self-propagation [1]. The best characterized yeast prion, [*PSI* ^+^], is the altered form of the translational release factor, Sup35, which forms large insoluble aggregates. Conformational conversion and sequestration of Sup35 into prion polymers limits the amount of natively folded Sup35 available for the translation process, resulting in a readily detectable nonsense suppression phenotype. The current model of *de novo* [*PSI* ^+^] formation suggests that conformationally altered Sup35 oligomers emerge in a spontaneous and Sup35-concentration dependent process [2]. In turn, the oligomeric species can act as a seed that is capable of nucleating the conformational conversion of non-prion polypeptides [3]. *In vivo*, the nucleation requirement is often fulfilled by another prion, [*PIN* ^+^], which is thought to enhance nucleation by cross-seeding [4], [5]. Propagation, defined as stable transmission of the prion trait to the progeny, involves [*PSI* ^+^] polymer elongation as well as fragmentation [6]. Thus, both emergence and propagation of the prion phenotype are constituted by protein folding and remodeling events, requiring the action of chaperones. Consequently, prion physiology is sensitive to changes in the cellular levels of certain chaperones or their regulatory factors.

The chaperone with the most prominent role in prion propagation is the AAA^+^-ATPase Hsp104 that functions to dissolve large protein aggregates and refold proteins [7], [8]. Hsp104 is essential for the propagation of all known yeast prions, as deletion or inactivation leads to formation of large aggregates that are not transmitted to the progeny, and likely represent dead-ends of the prion cycle [9], [10]. It is thought that the disaggregase activity of Hsp104 cleaves [*PSI* ^+^] aggregates into seeds, which can subsequently propagate the conversion of normal Sup35 [11], [12]. Interestingly, cells can also be cured of the [*PSI* ^+^] phenotype by Hsp104 overproduction [11], [13], perhaps through excessive fragmentation of [*PSI* ^+^] polymers which yields non-seeding Sup35 species [14]. However, mutational analysis has led to the suggestion that Hsp104-mediated curing of [*PSI* ^+^] may not solely be the result of aggregate fragmentation, but involves a distinct activity requiring additional functional domains of Hsp104 [15], [16]. As other prions are unaffected by increased levels of Hsp104, this may reflect a specific interaction with Sup35 [14].

The two classes of cytosolic Hsp70 chaperones in yeast, Ssa1-4 and Ssb1,2, also affect *de novo* prion formation as well as propagation, although with opposing effects. [*PSI* ^+^] formation induced upon Sup35 overexpression is stimulated by overexpression of Ssa or reduced levels of Ssb1 [17], [18], while propagation is inhibited by overexpression of Ssb1 or reduced levels of Ssa [19], [20]. Further, curing of [*PSI* ^+^] by Hsp104 overexpression is inhibited by Ssa1 overproduction, or by the absence of Ssb1 [2], [17], [18], [21]. It has been proposed that these opposing effects reflect the cellular roles of these chaperones; Ssa1 stabilizes misfolded proteins such that they can serve as substrates for prion conversion, while Ssb1 may stimulate the productive folding of newly synthesized proteins to the native state [17]. Thus, prion propagation and maintenance are sensitive to changes in Hsp70 activities.

The Hsp110 proteins, a sub-group of the Hsp70 superfamily, are represented by Sse1 and Sse2 in yeast. Their essential cellular role is illustrated by the impaired growth of *sse1Δ* cells and the lethal phenotype of *sse1Δ sse2Δ* cells [22], [23]. We and others recently reported that Sse1 serves as a nucleotide exchange factor (NEF) for members of both classes of cytosolic Hsp70 chaperones, Ssa1 and Ssb1 [23], [24]. The NEF function is the essential activity of Sse1 and Sse2 proteins, as overexpression of another NEF, Fes1, restores viability of the *sse1Δ sse2Δ* strain [23]. However, the structural similarity of Hsp110 proteins with Hsp70 chaperones, and particularly the existence of a putative substrate binding domain [25], [26], suggests that Hsp110 proteins may also be capable of binding substrate. Indeed, an *in vitro* holdase activity that prevents protein aggregation has been reported for Sse1 [27]. Thus, studying the role of Sse1 within the context of prion biology can provide a novel perspective of its precise function within the cytosolic Hsp70 chaperone network.

In agreement with recent reports [28], [29], Sse1 is required for efficient *de novo* [*PSI* ^+^] formation as well as for stable propagation of the [*PSI* ^+^] phenotype. The requirement is for the nucleotide exchange activity of Sse1, as overexpression of another NEF for Hsp70 chaperones, Snl1ΔN [30], can suppress *sse1Δ* deficiencies in [*PSI* ^+^] propagation. Moreover, Sse1 is more potent than Ssa1 in counteracting [*PSI* ^+^] curing by Hsp104 overproduction, suggesting a direct promotion of the Sup35 prion conformation by Sse1. In fact, Sse1 enables more efficient *in vitro* fiber assembly by decreasing the lag phase. Thus, Sse1 is a nucleotide exchange factor whose activity on the Hsp70 chaperone Ssa1 is central to the establishment and maintenance of [*PSI* ^+^]. In addition, Sse1 may promote prion conversion of Sup35 by directly stabilizing folding intermediates.

## Materials and Methods

### Assays for [*PSI* ^+^] formation and curing

The presence of [*PSI* ^+^] was assessed through the inability of the Sup35 prion form to terminate translation. The resulting read-through of the *ade1-14* (UGA) mutant allele enables adenine biosynthesis and abolishes the accumulation of a red pigment. Thus, [*psi* ^−^] and [*PSI* ^+^] cells grow as red or pink/white colonies on rich media, respectively [11]. The OT55 and OT56 strains differ in degree of nonsense suppression, yielding ‘weak’ or ‘strong’ [*PSI* ^+^] phenotypes, respectively [31]. Strains used in this study are listed in Table 1.

**Table 1:** Strains used in this study

Strain Name	[*PSI* ^+^] phenotype	Genotype	Reference
OT60	[*psi* ^−^ *PIN* ^+^]	74-D694 *(MAT* ***a*** * ade1-14(UGA) his3-Δ 200 leu2-3,112 trp1-289 ura3-52)*	[28]
GT17	[*psi* ^−^ *pin* ^−^]	74-D694	[16]
OT56	[*PSI* ^+^ *PIN* ^+^]_s_	74-D694	[28]
OT55	[*PSI* ^+^ *PIN* ^+^]_w_	74-D694	[28]
OT60 *sse1Δ*	[*psi* ^−^ *PIN* ^+^]	74-D694 *sse1Δ::KAN*	This study
OT56 *sse1Δ*	[*PSI* ^+^ *PIN* ^+^]	74-D694 *sse1Δ::KAN*	This study
OT55 *sse1Δ*	[*psi* ^−^ *PIN* ^+^]	74-D694 *sse1Δ::KAN*	This study
OT56 *sse1,2Δ SSE1*	[*PSI* ^+^ *PIN* ^+^]	74-D694 *sse1Δ::KAN sse2Δ::HIS pCUA-SSE1*	This study
GT81C	[*PSI* ^+^ *PIN* ^+^]_s_	*MATa ;ade1-14; his3-Δ200 or 11,15; leu2.3,112; lys2; trp1Δ; ura3-52*	[16]
GT81C *sse1Δ*	[*PSI* ^+^ *PIN* ^+^]	GT81C *sse1Δ::KAN*	This study
GT146	[*PSI* ^+^ *PIN* ^+^]_s_	GT81C *ssb1Δ::HIS ssb2Δ*	[16]
GT146 *sse1Δ*	[*PSI* ^+^ *PIN* ^+^]	GT146 *sse1Δ::KAN*	This study

Quantitation of Hsp104-mediated curing of [*PSI* ^+^] as well as the quantitative assessment of [*PSI* ^+^] formation upon Sup35 overexpression were performed as described [32].

Transformants were grown as suspension cultures in selective media, and expression of Hsp104 from pHG28 or Sup35 from pLA1-SUP35 was induced along with that of (co-)chaperones by inoculating log-phase cells into selective media containing 2% galactose and 2% raffinose. Ssa1 and Ssb1 were expressed from p316-GAL plasmids, while Sse1 and Snl1ΔN were expressed from p315-GAL plasmids (please refer to Table 2 for plasmids used in this study). After different induction periods, cells were plated onto selective media and [*PSI* ^+^] phenotypes were scored after replica plating onto rich media as well as media lacking adenine. Data presented are an average of at least three independent experiments with the standard error of the mean.

**Table 2:** Plasmids used in this study

Name	Type	Marker	Promoter-Expression cassette	Reference
pPROEX-Htb-SNL1ΔN	Protein expression		*HIS-SNL1ΔN*	This study
pLA1	CEN	HIS3	-	[17]
pHG28	CEN	HIS3	P*_GAL_*-*HSP104*	[16]
pLA1-SUP35	CEN	HIS3	P*_GAL_*-*SUP35*	[16]
pCUA-SSE1	CEN	URA3	P*_ADH_*-*SSE1*	This study
pRS316	CEN	URA3	-	[45]
pRS316-GAL-SUP35	CEN	URA3	P*_GAL_*-*SUP35*	This study
pRS316-GAL-SSA1	CEN	URA3	P*_GAL_*-*SSA1*	This study
pRS316-GAL-SSB1	CEN	URA3	P*_GAL_*-*SSB1*	This study
pRS315	CEN	LEU2	-	[45]
pRS315-GAL-SSE1	CEN	LEU2	P*_GAL_*-*SSE1*	This study
pRS315-GAL-SNL1ΔN	CEN	LEU2	P*_GAL_*-*SNL1ΔN*	This study
pRS315-GPD-SSA1	CEN	LEU2	P*_GPD_*-*SSA1*	This study
pRS315-GPD-SSB1	CEN	LEU2	P*_GPD_*-*SSB1*	This study
pRS315-GPD-SSE1	CEN	LEU2	P*_GPD_*-*SSE1*	This study
pRS315-GPD-SNL1ΔN	CEN	LEU2	P*_GPD_*-*SNL1ΔN*	This study
pRS425-GPD-FES1	2µ	LEU2	P*_GPD_*-*FES1*	This study
pRS425-GPD-SNL1ΔN	2µ	LEU2	P*_GPD_*-*SNL1ΔN*	This study
p2H-GPD-FES1	2µ	HIS3	P*_GPD_*-*FES1*	This study
p2H-GPD-SNL1ΔN	2µ	HIS3	P*_GPD_*-*SNL1ΔN*	This study

### Aggregation analyses of Sup35 and Rnq1

Cultures were grown in appropriate selective media to mid-log phase and protein lysates were prepared as described [32], [33]. Sup35 aggregates were separated with a low-speed spin (16,000×g, 20 min.), while Rnq1 and potential Sse1 aggregates were pelleted by ultra-centrifugation at 280,000×g, 30 min.

### Antibodies and protein analysis

Analysis of chaperone protein levels and centrifugation analyses of Sup35 and Rnq1 were performed by SDS-PAGE and Western Blotting using standard techniques. Analysis of polymeric Sup35 by semi-denaturing detergent-agarose gel electrophoresis was performed as described [34]. Polyclonal antibodies against Ssa1, Ssb1 and Sse1 were raised against purified proteins, as were the Rnq1 and Sup35 antibodies as described [33], [35]. The rabbit polyclonal antibody recognizing G6PDH was purchased from Sigma.

### Protein purification

Sse1 was expressed and purified as described previously [36]. The gene encoding Snl1 lacking the N-terminal transmembrane domain (1-39aa) (Snl1ΔN) was cloned into a pProEX Htb vector (Invitrogen) using standard procedures. Snl1ΔN was expressed with a TEV-cleavable N-terminal His6-tag in BL21 Star bacteria (Invitrogen) and purified by Ni-IDA affinity chromatography (Protino, Macherey-Nagel) following standard protocols. N-terminally GST-tagged Fes1 was expressed from the pGEX-4T-2 vector (Amersham Biosciences) and purified as described [37].

### Stopped-flow analysis:

Stopped-flow analysis was performed using an Applied Photophysics (Surrey, UK) SX-18MV instrument. 0.5 µM Ssa1 was pre-incubated with 0.5 µM MABA-ADP (N^8^-(4-N′-methylanthraniloylaminobutyl)-ADP) in HKM buffer (25 mM Hepes-KOH pH 7.6, 50 mM KCl, 5 mM MgCl_2_) for 30 minutes at 30°C [38]. For determination of dissociation rates, equal volumes of Ssa1-MABA-ADP complex and HKM buffer supplemented with 500 µM ATP with or without 2.5 µM nucleotide exchange factor were rapidly mixed in the stopped flow device and the decrease in fluorescent signal was monitored (time scales ranged from 0.1 to 10 sec). The kinetic rate constants were obtained by fitting the data using the Applied Photophysics SpectraKinetic Workstation v4.56-1.

### In vitro fiber assembly

Untagged Sup35NM was expressed in *E. coli* BL21[DE3] (pLysS) (Stratagene) and purified as described [12], [32]. Sup35NM fiber assembly was initiated by diluting 0.5 mM Sup35NM (in 20 mM TrisHCl pH 7.4, 8 M urea) to 2.5 µM with Sup35NM assembly buffer (NAB) (40 mM Hepes-KOH pH 7.4, 150 mM KCl, 20 mM MgCl_2_, 5 mM ATP, 1 mM DTT) plus ATP regeneration system as described [12]. All purified proteins added into the assembly reactions were exchanged into NAB, and were present upon resuspension of Sup35NM from denaturant. For the no-nucleotide condition, nucleotide and MgCl_2_ were omitted from NAB and NaEDTA (20 mM) was added.

## Results

### Sse1 NEF activity is critical for [*PSI* ^+^] propagation

Two recent studies have demonstrated that Sse1 is required for efficient propagation of [*PSI* ^+^] as *SSE1* deletion results in the loss of a weak [*PSI* ^+^] phenotype ([*PSI* ^+^]_w_) and impairment of a strong [*PSI* ^+^] phenotype ([*PSI* ^+^]_s_) [28], [29]. We examined these phenotypes under our experimental conditions, and confirmed that meiotic segregants lacking the *SSE1* gene exhibited a weakened prion phenotype in both [*PSI* ^+^]_w_ and [*PSI* ^+^]_s_ backgrounds (Fig. S1A, C). Further, centrifugation analysis of cellular lysates established that *sse1Δ* strains have an increased level of soluble Sup35 as compared to wild-type cells (Fig. S1B, compare lanes 8 and 11). This disruption of Sup35 aggregates did not reflect a general effect on all yeast prions, as the Rnq1 protein was still pelletable in the *sse1Δ* cells (Fig. S1B lower panel). These observations are consistent with a general impairment in [*PSI* ^+^] propagation in the *sse1Δ* strains.

**Figure 1:**
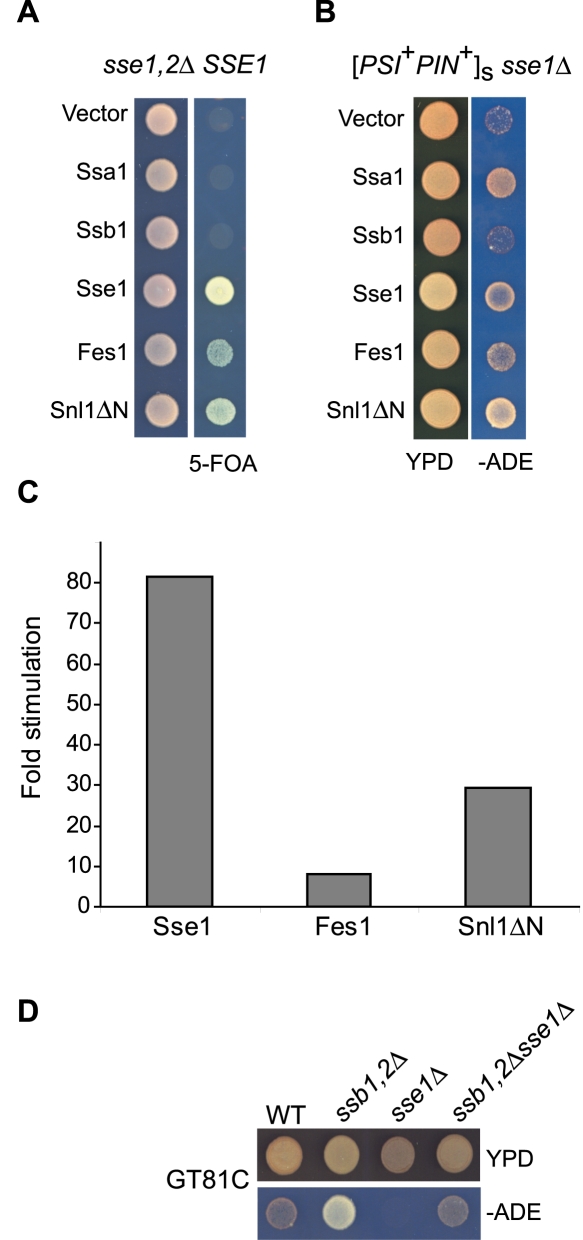
Nucleotide exchange by Sse1 is required for efficient [*PSI* ^+^] propagation. (A) Complementation of the *sse1Δ sse2Δ* double deletion by overexpression of chaperones or nucleotide exchange factors. *sse1Δ sse2Δ* cells expressing Sse1 from pCUA-SSE1 were transformed with plasmids encoding chaperones or nucleotide exchange factors under control of the GPD promotor. Ssa1, Ssb1 and Sse1 were expressed from p315-GPD-plasmids, while Fes1 and Snl1ΔN were expressed from p425-GPD-plasmids. Transformants were spotted onto selective media (left panel) or media containing 5-FOA (right panel) to select against the SSE1 plasmid. (B) Complementation of the [*PSI* ^+^]_s_ phenotype of *sse1Δ* cells. [*PSI* ^+^]_s_ *sse1Δ* cells were transformed with plasmids encoding chaperones or nucleotide exchange factors from p315-GPD-plasmids (Ssa1, Ssb1 and Sse1) or p2H-GPD-plasmids (Fes1 and Snl1ΔN) and [*PSI* ^+^] phenotypes were assessed by growth on rich media (YPD) after 2 days and media lacking adenine (-Ade) after 3–6 days. (C) Nucleotide exchange activities of Sse1, Fes1, and Snl1ΔN on Ssa1. NEF activities of a 5-fold excess of nucleotide exchange factors (2.5 µM) over the Hsp70 chaperone Ssa1 (0.5 µM) were determined using stopped-flow analysis. Determined k_off_ values in the presence of NEF were related to the basal k_off_ for release of MABA-ADP from Ssa1 to yield the fold stimulation. (D) [*PSI* ^+^] phenotype of *sse1Δssb1,2Δ* cells. [*PSI* ^+^] *sse1Δ* cells were crossed with the corresponding [*PSI* ^+^] *ssb1Δ ssb2Δ* cells. Diploids were sporulated and [*PSI* ^+^] phenotypes of segregants were assessed as stated above.

We sought to define the precise activity of Sse1 required for prion propagation. As Sse1 is a potent NEF for both the Ssa and the Ssb classes of Hsp70s, this is putatively the key activity for prion propagation. Alternatively, Sse1 may execute chaperone-like functions within the cell that support the maintenance of [*PSI* ^+^]. While others have proposed that a potential chaperone-like activity of Sse1 is not involved in [*PSI* ^+^] formation [29], the nucleotide binding (G233D) and hydrolysis (K69Q) mutants used do not exclude a putative holdase activity and they do not directly address the role of the NEF activity in this process. We approached this issue by replacing Sse1 with two other NEF proteins. First, we constructed an *sse1Δ sse2Δ* strain expressing Sse1 from a *URA3* marked plasmid. Viability of this strain is dependent on the plasmid-encoded Sse1, as evident by the lack of growth when cells were plated on 5-FOA, a Ura^+^ counter-selection medium (Fig. 1A). Other NEFs can replace the essential role of Sse proteins, since both overexpression of Fes1 or the catalytic domain of Snl1 (Snl1ΔN) [30] supported strain growth. In contrast, upregulation of either Ssa1 or Ssb1 was insufficient to restore viability, indicating that any potential chaperone-like role of Sse1 is secondary to its NEF activity.

Having established that overexpression of either Fes1 or Snl1ΔΝ is sufficient to replace the essential activity of Sse1, we directly tested if these NEFs could replace Sse1 in prion propagation. The [*PSI* ^+^] phenotype was assessed by color development on rich medium and growth on adenine-deficient medium. In this assay, [*PSI* ^+^] is scored as nonsense suppression of the *ade1-14* adenine auxotrophy allele, resulting in colonies that can grow on media lacking adenine and appear pink or white on rich media (see Materials & Methods). A [*PSI* ^+^]_s_ *sse1Δ* strain grows poorly on adenine-deficient media due to a weakened nonsense suppression resulting from the *sse1Δ* (Fig. 1B). However, overexpression of Sse1, Fes1 or Snl1ΔN improved growth of the strain under adenine-limiting conditions, indicating that prion propagation does not appear to specifically require Sse1 but a general increase in NEF levels.

An NEF functions to catalyze the ADP to ATP exchange of Hsp70s, resulting in substrate release from the chaperone [24]. This raises the possibility that *sse1Δ* cells are impaired in prion propagation due to limiting amounts of free Hsp70. We tested this by overexpressing Ssa1 in the [*PSI* ^+^]_s_ *sse1Δ* strain and indeed found that the impaired prion propagation was alleviated (Fig. 1B). Thus, propagation appears to not require Sse1 specifically, but rather an increased level of free Ssa1 in the cytosol; a state which can also be achieved with upregulation of an NEF. The correlation between NEF levels and substrate binding by Hsp70s has been demonstrated by Yam et al. [39], who observed that higher levels of Ssa and Ssb in *sse1Δ* cells remain bound to substrate polypeptides.

We considered that differences in the degree of complementation seen by Sse1, Fes1 and Snl1ΔN in the [*PSI* ^+^]_s_ *sse1Δ* strain might be due to their respective nucleotide exchange activities. Thus, we measured nucleotide exchange rates by stopped flow analysis using a 5-fold excess of NEF to Ssa1 (Fig. 1C). In agreement with previous reports [23], [24], Sse1 is a potent NEF for Ssa1 that accelerates the basal ADP release rate about 81-fold (k_off_ = 40 s^−1^). Fes1 accelerates nucleotide release only 8-fold (k_off_ = 4 s^−1^). We find that Snl1ΔN possesses an intermediate activity, stimulating nucleotide release about 30-fold (k_off_ = 14 s^−1^). Furthermore, Snl1ΔN is more effective than Fes1 in its ability to stimulate nucleotide release from Ssb1 (data not shown). The higher NEF activity of Snl1ΔN as compared to Fes1 provides an explanation for the more efficient suppression of the *SSE1* deletion (Fig 1B). Thus, in further experiments we used Snl1ΔN as a control for nucleotide exchange activities.

### Sse1 antagonizes Ssb in prion propagation

While Sse1 is a NEF for both Hsp70 chaperones, Ssa1 and Ssb1, *in vitro* [23], [24], it is uncertain how this interplay is regulated within the cell. Ssa1 and Ssb1 affect prion propagation differently and therefore provide a means to delineate the relationships between Sse1 and the Hsp70s. Double deletion of *ssb1* and *ssb2* in a [*PSI* ^+^]_s_ strain leads to increased nonsense suppression, consistent with an antagonistic influence of Ssb1,2 on the [*PSI* ^+^] state [18] (Fig. 1D). We examined the [*PSI* ^+^] phenotypes of progeny from crosses between the *sse1Δ* strain and the *ssb1Δssb2Δ* strain. Cells with the triple deletions *sse1Δ ssb1Δ ssb2Δ* exhibited a [*PSI* ^+^] phenotype that was stronger than for *sse1Δ* cells, yet weaker than for *ssb1Δ ssb2Δ* cells. Thus, the reduced nonsense suppression of *sse1Δ* cells appears to be counteracted by the *ssb1Δ ssb2Δ* double deletion. Taken together with the observation that Ssa1, but not Ssb1 overexpression, suppresses the prion propagation defect of *sse1*Δ cells, the data suggest that Sse1 mainly functions together with Ssa1.

### Increased NEF activity increases efficiency of [*PSI* ^+^] formation

**Figure 2:**
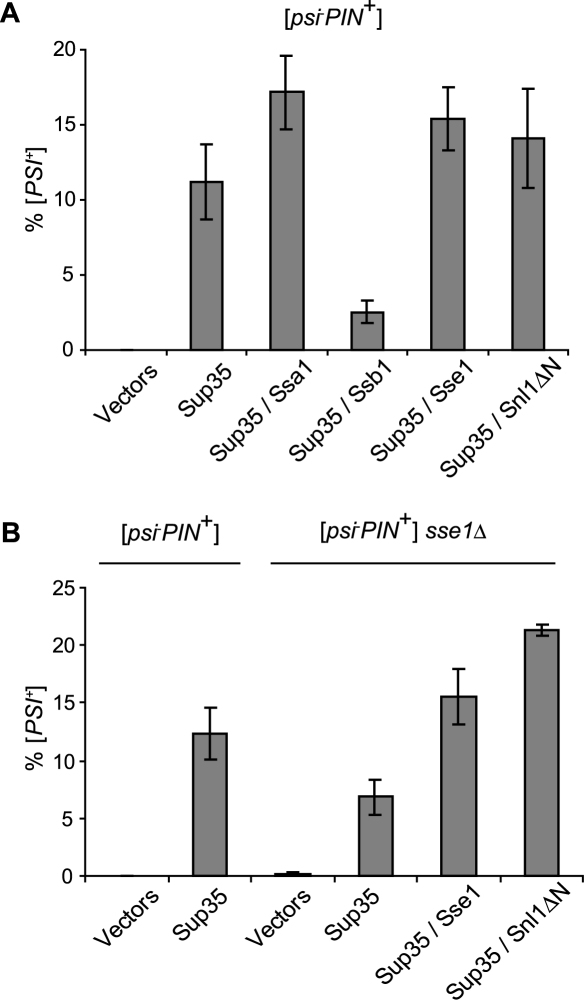
Nucleotide exchange by Sse1 is important for *de novo* [*PSI* ^+^] formation. (A) Quantitation of [*PSI* ^+^] formation upon overexpression of (co-)chaperones. [*psi* ^−^ *PIN* ^+^] cells were transformed with pLA1-SUP35 and p316-GAL-SSA1, p316-GAL-SSB1, p315-SSE1 or p315-GAL-SNL1ΔN. Transformants were grown as suspension cultures in selective media and expression of Sup35 along with (co-)chaperones was induced with galactose for 2 days. Cells were plated onto selective media and [*PSI* ^+^] phenotypes were scored after replica plating onto rich media as well as media lacking adenine. Error bars represent the standard error of the mean. (B) Quantitation of [*PSI* ^+^] formation in wild-type vs. *sse1Δ* cells. [*psi* ^−^ *PIN* ^+^] wild-type or *sse1Δ* cells were transformed with pLA1-SUP35 expressing Sup35 under control of the GAL promotor, as well as with p315-GPD-SSE1 or –SNL1ΔN. Quantitation of [*PSI* ^+^] induction was performed as stated above. Error bars represent the standard error of the mean.

Aside from its role in propagation of [*PSI* ^+^], Sse1 has recently been demonstrated to be involved in [*PSI* ^+^] formation [29]. We examined whether it is the NEF activity of Sse1 that promotes [*PSI* ^+^] formation. As seen by others [17], co-expression of Ssa1 with Sup35 increased [*PSI* ^+^] formation as compared to Sup35 alone (Fig. 2A). Ssb1 suppressed conversion to the [*PSI* ^+^] state by a factor of about 4, consistent with the antagonistic effects of higher Ssb1 levels in prion propagation [19]. Similar to Ssa1, co-expression of either Sse1 or Snl1ΔN with Sup35 had a modest positive effect on [*PSI* ^+^] formation, resulting in an approximately 1.4 fold or 1.2 fold increase in [*PSI* ^+^] cells, respectively, as compared to overexpression of Sup35 alone (Fig. 2A) [29]. Thus, while Ssb1 upregulation consistently antagonizes [*PSI* ^+^] formation, increased levels of NEF mimic the ability of Ssa1 to stimulate prion formation. Analysis of [*PSI* ^+^] induction by Sup35 overexpression in [*psi* ^−^ *PIN* ^+^] *sse1Δ* cells confirmed previous observations that [*PSI* ^+^] is induced to only 50% of the level achievable in wild type cells (Fig. 2B) [29]. This suggests that Sse1 is required for efficient [*PSI* ^+^] formation or propagation once the seed is formed. Importantly, this deficiency was suppressed with constitutive co-expression of either Sse1 or Snl1ΔN (Fig. 2B). These findings, in addition to confirming the recent observation that Sse1 is required for efficient [*PSI* ^+^] formation [29], indicate that the NEF activity alone is important in this process.

### Sse1 strongly inhibits Hsp104-mediated curing of [*PSI* ^+^]

**Figure 3:**
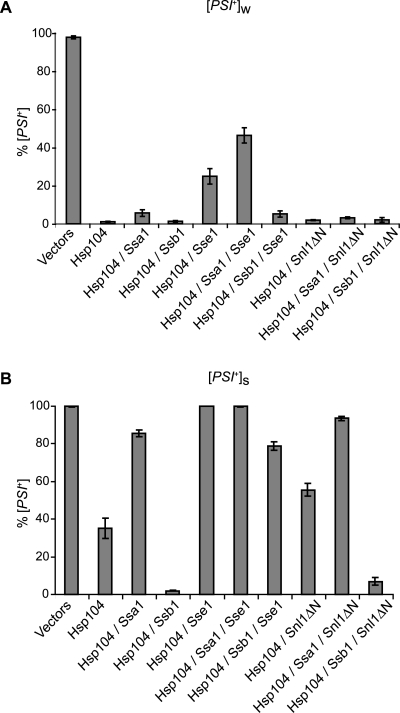
Hsp104 mediated curing of [*PSI* ^+^]. (A) Quantitation of [*PSI* ^+^] curing by Hsp104 overexpression in a [*PSI* ^+^]_w_ strain. [*PSI* ^+^]_w_ cells were transformed with pHG28 for expression of Hsp104, as well as with p316-GAL-SSA1, p316-GAL-SSB1, p315-SSE1 or p315-GAL-SNL1ΔN and combinations thereof. Transformants were grown as suspension cultures in selective media and expression of Hsp104 along with (co-)chaperones was induced with galactose for 1 day. Cells were plated onto selective media and [*PSI* ^+^] phenotypes were scored after replica plating onto rich media as well as media lacking adenine. Error bars represent the standard error of the mean. (B) Quantitation of [*PSI* ^+^] curing by Hsp104 overexpression in a [*PSI* ^+^]_s_ strain. Quantitation of Hsp104 mediated-curing was performed as stated above.

Sse1 appears to influence prion formation and propagation by preferentially stimulating Ssa1 over Ssb1, however, another aspect of propagation to consider is the polymer fragmentation mediated by Hsp104. The cycle of [*PSI* ^+^] propagation is extremely sensitive to Hsp104 levels, such that up- or downregulation of the chaperone leads to curing of [*PSI* ^+^] [11]. Furthermore, overexpression of Ssa1 and Ssb1 have strikingly different effects on the curing of [*PSI* ^+^] mediated by Hsp104 overexpression. We quantitated the effect of Ssa1, Ssb1, Sse1 and Snl1ΔN overexpression on Hsp104-mediated curing of both a weak and a strong [*PSI* ^+^] strain (Fig. 3A, B). Chaperone expression from centromeric plasmids was induced with galactose for 24 hours, and the [*PSI* ^+^] status was assessed. As previously observed, upregulation of Ssa1 suppresses the curative effect of Hsp104 overexpression [17], [21], and directly contrasts with the increased curing of [*PSI* ^+^] cells when Ssb1 is co-overexpressed [18], [19]. Strikingly, the combined upregulation of Sse1 and Hsp104 resulted in a marked resistance to curing as compared to overexpression of Hsp104 alone. Thus, Sse1 suppresses the curative abilities of Hsp104 similar to Ssa1, although the effect of Sse1 was dramatically greater; this is most evident in the weak strain.

The increased effectiveness of Sse1 over Ssa1 in inhibiting Hsp104-mediated curing may be the result of an insufficient endogenous NEF activity that is rate-limiting for Hsp70 activities. If this is the case, then upregulation of an NEF would overcome these limitations, both for Ssa1 and Ssb1. Indeed, combined overexpression of Ssa1 and Sse1 further inhibited curing, although the co-expression of both Sse1 and Ssb1 lessened the increase in Hsp104-mediated curing as compared to Ssb1 alone. The latter finding is consistent with a closer working relationship of Sse1 with Ssa1, such that any stimulation of Ssb1 may be overshadowed by the more dominant Ssa1 activities. This hypothesis is not consistent, however, with the marginal influence of Snl1ΔN on either Ssa1 or Ssb1 effects, or the fact that in previous assays (i.e. [*PSI* ^+^] formation (Fig. 2)), Ssa1 activities were similar or greater than those observed for Sse1 and did not appear limited by endogenous NEF levels. Therefore, an alternative interpretation for the increased effectiveness of Sse1 over Ssa1 in inhibiting Hsp104-mediated curing is that Sse1 somehow promotes the Sup35 prion conformation; this activity would compete with the continuous destruction of seeds and facilitate propagation of prions rather than their loss.

### Sse1 stimulates *de novo* Sup35NM fiber assembly *in vitro*

**Figure 4:**
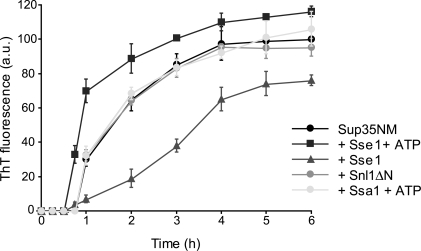
Sse1 stimulates spontaneous Sup35NM fibre assembly. Kinetics of rotated (80 rpm) Sup35NM (2.5 µM) fibre assembly in the absence or presence of Snl1ΔN (2.5 µM), Sse1 (2.5 µM) plus ATP (5 mM), Sse1 (2.5 µM) in the absence of nucleotide, or Ssa1 (2.5 µM) plus ATP (5 mM). Fiber assembly was determined by thioflavin T fluorescence. Error bars represent the standard deviation.

To directly evaluate the possibility that Sse1 may promote the Sup35 prion conformation, we examined the kinetics of Sup35NM *in vitro* fiber assembly. This system has been used to delineate the precise role of Hsp104-mediated effects on different stages of prion assembly and disassembly [12], [14]. The results obtained with the NM fragment, consisting of the N-terminal and middle region of Sup35 protein, are similar to those for the full-length protein, however, complicating folding and chaperone sequestering effects of the C-terminal domain are avoided. Sup35NM assembles into fibers after a lag phase of 45–60 min and fiber assembly is complete after ∼6 hrs as determined by thioflavin-T fluorescence or acquisition of SDS-resistance (Fig. 4, data not shown)

When Sse1 was included at equimolar levels into the fiber assembly reaction, a significant stimulatory effect was observed. The lag phase was decreased such that assembly was ∼30% complete after 45 min, whereas no assembly had yet occurred in reactions containing Sup35NM alone. This stimulatory effect was not observed when Sup35NM assembly was seeded with preformed Sup35NM fibers (data not shown), indicating that Sse1 stimulated the initial nucleation of Sup35NM fibers rather than an elongation step. Furthermore, stimulatory effects were not observed with Ssa1 or other Hsp70 s (data not shown), nor another NEF, Snl1ΔN. The ability of Sse1 to stimulate fiber formation was dependent on the presence of ATP. This is somewhat unsurprising, as Sse1 in the ATP-state has a more compact conformation as compared to the structurally flexible nucleotide-free state [36], [40]. In the absence of nucleotide, Sse1 delayed Sup35NM fiber assembly; the flexible nucleotide-free form of Sse1 may interact non-specifically with Sup35NM, thereby impeding prion nucleation.

### Sse1 and Ssa1 can induce [*PSI* ^+^] formation in the absence of [*PIN* ^+^]

**Figure 5:**
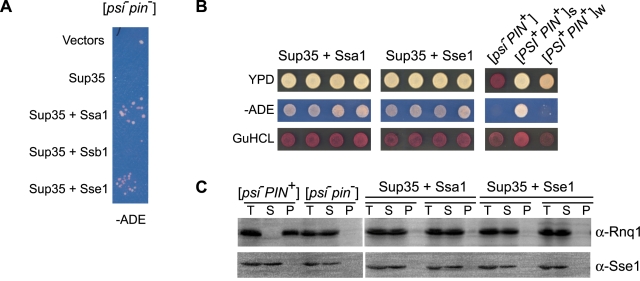
[*PSI* ^+^] formation in [*pin* ^−^] cells. (A) [*psi* ^−^ *pin* ^−^] cells were transformed with pLA1-SUP35 and p316-GAL-SSA1, p316-GAL-SSB1 or p315-SSE1. Transformants were grown on selective media and expression was induced by replica plating onto selective media containing galactose. After 8 days of induction, plates were replicated onto media lacking adenine and growth was assessed after incubation at 30°C for 13 days. (B) Verification of [*PSI* ^+^] induction in [*pin* ^−^] cells. Papillae from adenine-deficient media were streaked to single colonies and the [*PSI* ^+^] phenotype was verified by growth on rich media and media lacking adenine as well as by loss of the nonsense suppression phenotype on media containing 3 mM guanidinium hydrochloride. (C) Centrifugation analysis of Rnq1 and Sse1 from [*PSI* ^+^] cells. Aggregation of Rnq1 and Sse1 in [*PSI* ^+^] cells was assessed by centrifugation analysis and Western Blotting.

One possible explanation for the ability of Sse1 to stimulate spontaneous Sup35NM fiber assembly is an ability to promote the folding events that nucleate Sup35NM fibers. Such an activity might facilitate [*PSI* ^+^] induction even in the absence of [*PIN* ^+^]. To address this possibility we examined whether the ability of Sse1 to induce [*PSI* ^+^] formation is dependent upon the [*PIN* ^+^] state of Rnq1. Others have observed a Rnq1-independent induction of [*PSI* ^+^], however, this only occurred with overexpression of another Q/N rich domain [5]. Remarkably, after an induction period of 8 days followed by 2 weeks of selection on adenine-lacking media, papillae were observed upon overexpression of Sse1 together with Sup35, but not in the control (Fig. 5A). Surprisingly, overexpression of Ssa1 and Sup35 caused the same effect (Fig. 5A). A previous study did not observe [*PSI* ^+^] induction upon Ssa1 overexpression [17], however, this is likely due to the shorter period of selection on adenine-deficient media normally employed. Cells from the papillae demonstrated the classical signs of a [*PSI* ^+^] phenotype, including strong growth on media lacking adenine, and curability by GuHCl-treatment (Fig. 5B). However, they remain [*pin* ^−^] as observed by a lack of Rnq1 aggregation (Fig 5C). Furthermore, these cells, once cured of [*PSI* ^+^] by Hsp104 overexpression, were unable to form [*PSI* ^+^] upon Sup35 overexpression (data not shown). Importantly, the *de novo* induction of [*PSI* ^+^] does not appear to result from a self-seeding tendency of Sse1, as these cells showed no difference in the level of pelletable Sse1 reactive material after ultra-centrifugation as compared to [*pin* ^−^] strains (Fig. 5C). Thus, overexpression of Ssa1 or Sse1 with Sup35 can obviate the requirement for [*PIN^+^*] in *de novo* [*PSI* ^+^] induction.

## Discussion

The influences of the cellular chaperone network on prion propagation are intricate and not well understood. An extra level of complexity has been added with the identification of the yeast Hsp110, Sse1, as a NEF for the cytosolic Hsp70 family members [23], [24]. Recent reports have suggested that Sse1 has a significant role in prion propagation [28], [29]. Our data support and extend these conclusions, and moreover, we suggest that Sse1 has additional NEF-independent activities that facilitate *de novo* seed formation.

The current models of the prion propagation cycle, based mainly on studies of the [*PSI* ^+^] prion, involve growth and division phases that are dependent on nucleating seeds. The growth phase comprises the binding and conversion of monomers by the prion seeds, resulting in fiber elongation [41]. The division phase is constituted by severing of the prion fibers by Hsp104, possibly with the assistance of Ssa1, so that there is a continuous supply of seeds for elongation [7], [8], [11], [12], [14]. If changes in chaperone levels result in excessive fiber growth without division, then the prion is unable to segregate evenly into the daughter cells, and is lost during cell division [9], [17].

One would anticipate that the ability of Sse1 to act as a NEF for the Hsp70 members would influence prion propagation, if only by facilitating Sup35 release from Ssa1 and Ssb1. Consistent with recent studies [28], [29] we show that cells lacking Sse1 exhibit a weakened prion state. We elucidate the mechanism behind this phenomenon by providing novel evidence that the levels of substrate-free Ssa1 are critical to prion propagation. Specifically, we demonstrate that upregulation of either another NEF or Ssa1 is sufficient to restore nonsense suppression in a strong [*PSI* ^+^] strain (Fig. 1B). Furthermore, we observe similar effects on prion formation when Sse1 or the NEF Snl1ΔN are overexpressed (Fig. 2). In contrast to our observations, Fan *et al*. [29] report that Sse1 stimulates prion formation more effectively than Ssa1. There are several notable experimental differences between the studies which are likely to impact on the final results; a primary one being that we, in contrast to Fan et al., used full-length Sup35 rather than the NM fragment. In conclusion, it is clear that Ssa1 levels play a central role in regulating prion propagation, and NEFs are critical in the regulation of substrate (i.e. Sup35) release from Ssa1 [39].

Our data also suggest that Sse1 influences prion propagation in an NEF-independent manner. First, Sse1 activities differ from those of Snl1ΔN in the inhibition of Hsp104-mediated curing. Second, Sse1 directly affects fiber assembly in an *in vitro* system. This remarkable feat of significantly decreasing the lag phase of *in vitro* fiber assembly has previously only been observed for Hsp104 [12]. While the precise mechanism is unclear, it is thought that Hsp104 accelerates the formation of obligate folding intermediates that nucleate the first seeds which are then elongated into fibers. As Sse1 is structurally related to the Hsp70 chaperone family [26] and was initially proposed to be a holdase [27], we speculate that Sse1 binds and stabilizes Sup35 folding intermediates. Such an activity is specific for folded and active Sse1, as the nucleotide dependence of Sse1 on fiber assembly can be directly correlated to the folding state of the protein [23], [40].

The primary difference between the experiments where Sse1 appears to exert a NEF-independent role, and those where only a NEF is required, is that the latter situations involve Sup35 overexpression. Overexpression has been used extensively to study [*PSI* ^+^] formation as it increases the frequency of nucleation sufficiently for experimental quantitation. However, this bears the risk that nucleation ceases to be a limiting factor [2]. Thus, we believe that under these conditions, any nucleation-promoting activity of Sse1 will be marginalized by the increase in spontaneous seed formation among an artificially expanded pool of available Sup35. This is supported by the observation that both Ssa1 and Sse1 can induce [*PSI* ^+^] formation in [*pin* ^−^] cells overexpressing Sup35 (Fig. 5). Even in the absence of [*PIN* ^+^], Sup35 overexpression appears to generate sufficient seeds for elongation and propagation by high levels of free Ssa1. Similarly, propagation of pre-existing [*PSI* ^+^] does not require *de novo* seed formation, and accordingly, we observe a requirement for only the NEF activity of Sse1 in this setting. In contrast to formation and propagation of [*PSI* ^+^], however, Hsp104-mediated curing may represent a situation where *de novo* seed formation is more relevant. The final Sup35 species generated by the Hsp104-mediated curing process cannot seed *in vitro* polymer formation [14]. One can speculate that Sse1 may act as a holdase to stabilize obligate intermediates of the Sup35 prion conformation and thereby facilitate seed formation. Thus, *in vivo*, overexpression of Sse1 would accelerate the prion formation step so efficiently as to outcompete the Hsp104-mediated disaggregation process.

Alternatively, Sse1 may act by directly inhibiting Hsp104 fiber severing activities, a mechanism that has been proposed for Ssa1-21 mediated impairment of the [*PSI* ^+^] phenotype [42], [43]. Genetic evidence indicates that the *SSA1-21* allele has an enhanced substrate binding activity that supports normal growth, but interferes with [*PSI* ^+^] propagation [44]. The avid binding of Ssa1-21 to Sup35 polymers has been suggested to directly impair Hsp104-mediated [*PSI* ^+^] remodelling by sterically restricting Hsp104 access [42], [43]. A similar hypothesis has been proposed for the observation that high levels of Ssa1 inhibit Hsp104 curing [21], and correlates with our and others observations (Fig. S1C) [17], that *SSE1* deletion or high Ssa1 levels increase polymer size. Correspondingly, an affinity of Sse1 for the prion conformation of Sup35 would explain our observations; association of Sse1 with obligate [*PSI* ^+^] conformational intermediates promotes conversion to the prion state, while binding of Sse1 to existing [*PSI* ^+^] polymers would sterically hinder the curing by Hsp104 overproduction.

In conclusion, we propose that Sse1 primarily influences prion propagation as an NEF for Hsp70, but has an additional novel role in stabilizing folding intermediates during *de novo* prion formation. This activity is relevant in the context of spontaneous [*PSI* ^+^] formation (i.e. in the absence of Sup35 overexpression). In addition, the potent inhibition of Hsp104-mediated curing by Sse1 may account for prion stability upon physiological Hsp104 induction; a role also proposed for Ssa1 [21]. Our results highlight the limitations of our understanding of the molecular processes involved at each step of the prion propagation cycle. Clearly, there remains much to be done to fully understand the complex *in vivo* dynamics underlying the process of prion formation and propagation.

## Supporting Information

Figure S1 Deletion of *SSE1* impairs [*PSI* ^+^] propagation. (A) Tetrad analysis of [*PSI* ^+^] *sse1Δ* cross with [*PSI* ^+^] wild-type cells. Wild-type cells of the weak ([*PSI* ^+^]_w_) or strong ([*PSI* ^+^]_s_) phenotypes were crossed with the corresponding [*PSI* ^+^] *sse1Δ* cells. Diploids were sporulated and [*PSI* ^+^] phenotypes of segregants were assessed by growth on rich media (YPD) after 2 days and media lacking adenine (-Ade) after 3–6 days. Upper panels: [*PSI* ^+^]_w_ tetrad. Lower panels: [*PSI* ^+^]_s_ tetrad. (B) Centrifugation analysis of Sup35 and Rnq1. Aggregation of Sup35 and Rnq1 in [*PSI* ^+^]_w_ wild-type and *sse1Δ* cells was assessed by centrifugation analysis and Western Blotting. (C) Agarose gel electrophoresis of Sup35 monomers and polymers. Monomeric and polymer forms of Sup35 in different strains were analyzed using SDD-AGE (semi-denaturing detergent-agarose gel electrophoresis) and Western Blotting. (D) Chaperone protein levels in *sse1Δ* cells or cells overexpressing Sse1. Levels of the chaperones Hsp104, Ssa1 and Ssb1 were determined by Western Blotting using G6PDH as a loading control.
(5.86 MB EPS)
